# High Density LD-Based Structural Variations Analysis in Cattle Genome

**DOI:** 10.1371/journal.pone.0103046

**Published:** 2014-07-22

**Authors:** Ricardo Salomon-Torres, Lakshmi K. Matukumalli, Curtis P. Van Tassell, Carlos Villa-Angulo, Víctor M. Gonzalez-Vizcarra, Rafael Villa-Angulo

**Affiliations:** 1 Laboratory of Bioinformatics and Biofotonics, Engineering Institute, Autonomous University of Baja California, Baja California, Mexico; 2 Animal Breeding, Genetics and Genomics at USDA, National Institute of Food and Agriculture (NIFA), Washington, District of Columbia, United States of America; 3 USDA-ARS, Bovine Functional Genomics Laboratories, Beltsville, Maryland, United States of America; 4 Veterinary Science Research Institute, Autonomous University of Baja California, Baja California, Mexico; Wageningen UR Livestock Research, Netherlands

## Abstract

Genomic structural variations represent an important source of genetic variation in mammal genomes, thus, they are commonly related to phenotypic expressions. In this work, ∼770,000 single nucleotide polymorphism genotypes from 506 animals from 19 cattle breeds were analyzed. A simple LD-based structural variation was defined, and a genome-wide analysis was performed. After applying some quality control filters, for each breed and each chromosome we calculated the linkage disequilibrium (*r* **^2^**) of short range (≤100 Kb). We sorted SNP pairs by distance and obtained a set of LD means (called the expected means) using bins of 5 Kb. We identified 15,246 segments of at least 1 Kb, among the 19 breeds, consisting of sets of at least 3 adjacent SNPs so that, for each SNP, *r* **^2^** within its neighbors in a 100 Kb range, to the right side of that SNP, were all bigger than, or all smaller than, the corresponding expected mean, and their *P-value* were significant after a Benjamini-Hochberg multiple testing correction. In addition, to account just for homogeneously distributed regions we considered only SNPs having at least 15 SNP neighbors within 100 Kb. We defined such segments as structural variations. By grouping all variations across all animals in the sample we defined 9,146 regions, involving a total of 53,137 SNPs; representing the 6.40% (160.98 Mb) from the bovine genome. The identified structural variations covered 3,109 genes. Clustering analysis showed the relatedness of breeds given the geographic region in which they are evolving. In summary, we present an analysis of structural variations based on the deviation of the expected short range LD between SNPs in the bovine genome. With an intuitive and simple definition based only on SNPs data it was possible to discern closeness of breeds due to grouping by geographic region in which they are evolving.

## Introduction

It is well known that among human beings, 99.9% of DNA sequence is identical [1], but, differences contribute to genetic variations among people. Early studies on the human genome were limited to those that could be identified through a microscope. Such variations were defined as structural variations with length of approximately 3 Mb or longer. As technologies evolved, scientists were able to characterize smaller differences, as well as abundant alterations in short DNA sequences, typically smaller than 1 Kb. In recent years, comparative sequence analyses have revealed DNA variation that involves segments that are smaller than those recognized microscopically, but larger than those readily detected by conventional sequence analysis. These variations range from ∼1 Kb to 3 Mb and consist on insertions, deletions, inversions and duplications and may even contain whole genes [2]. The impact of these variations can range from no observable difference to gene interruption. A structural variation is formally defined as a genomic alteration involving DNA segments larger than 1 Kb. Changes of this magnitude range from submicroscopic to microscopic. A structural variation is designated as structural abnormality when it is established that it can, by itself, or in combination with other environmental factors, be the cause of a genetic disease or a phenotypic expression.

Structural variations based on genotyping data have recently been the focus of interest, especially of the Copy Number Variation (CNV) type. The most used approach for detection of CNVs is based on two measures of signal intensity in each SNP: the Log R ratio (a normalized measure of the total signal intensity for two alleles of the SNP), and the B Allele Frequency (a normalized measure of the allelic intensity ratio of two alleles). The combination of Log ratio and B Allele Frequency is used to infer copy number changes in the genome [3], [4]. Other studies have been focused on the feasibility of linkage disequilibrium (LD) for CNV detection [5]–[7]. A study comparing LD from common haplotype regions between different populations [7] reported that high LD is not always a signal for a genomic variation, and low LD can be implicated in insertions and deletions. Another study of LD between SNPs and CNVs revealed that traditional LD measures are sufficient for SNPs outside the CNV, however the same metrics inappropriately quantify the covariance for SNPs inside the CNVs [6]. In a recent study, deletion type CNVs on bovine chromosome 6 was predicted from its neighboring SNP with a multiple regression model, by *Kadri et al.* (2012) [8]. They conclude that the genotype of a deletion type CNV and its putative QTL effect can be predicted with a maximum accuracy of 0.94 from surrounding SNPs. This high prediction accuracy suggests that genetic variation due to simple deletion CNV is well captured by dense SNP panels. Structural variations have been the focus of a number of recent studies in Cattle genome, especially CNVs [9]–[17]. However variations of other type rather than CNVs have not been analyzed. Using high density of SNP markers evenly distributed in the genome it is possible to detect regions with significant LD deviation compared to the expected value, which can be interpreted as short range genomic variation, and could help in future studies for assessing association with other type of Structural Variations.

Illumina, Inc, in collaboration with bovine researchers has developed the BovineHD panel of 777,962 SNPs genome-wide, making it the highest density panel in livestock. *Matukumalli et al (2011)* [18], genotyped a group of over 500 animals from 19 breeds and analyzed LD blocks and CNV segments. In this article, we used these data to inspect the distribution of structural variations based on the short-range linkage disequilibrium (LD) patterns genome-wide for all the breeds in the sample. First, we present an analysis of the average decline of the short-range LD across different distances; then, we propose a definition of structural variation based on the average short-range LD pattern, and we examine the whole genome to study the distribution of these variations. Clustering methods allowed us to differentiate between breed groups based on structural variations statistics. Finally, we reviewed the coincidences in the structural variations obtained with our method and different types of variations previously reported.

## Materials and Methods

### Animal Samples and data description

A set of 777,962 SNPs assayed by the BovineHD Genotyping BeadChip was provided by the International Bovine HapMap Consortium [19]. The SNPs were mapped in the UMD3.1 assembly. There were 506 animals in the sample. The animals were sampled from 19 different breeds (for details see Table S1 in File S1). All breeds belong to the *Taurus and Indicus* subspecies of the *Bos taurus* species. We grouped them by purpose and geographical origin as follows: British meat breeds are the Angus, Hereford and Red Angus. European meat breeds are Charolais, Limousin, Piedmontese and Romagnola. Dairy British breeds are Guernsey and Jersey. European dairy breeds are Brown Swiss, Holstein and Norwegian Red. The *Indicus* group includes the Brahman, Nelore and Gir breeds. The African breeds are formed by the N'Dama and Sheko and lastly, the hybrid breeds (*Taurus x Indicus*) are formed by the Santa Gertrudis and BeefMaster.

### Quality Control filters

Quality control filters were applied in order to eliminate technology errors of the “no call” type, to eliminate all the genotypes that violated the Hardy-Weinberg equilibrium, and monomorphic markers (markers with MAF<0.05% were also eliminated). After these quality control filters we guarantee a global quality over all the samples and we worked, in average between the 19 breeds, with a total of 523,651 markers which represents 67.31% of the initial information (for more details see Table S2 in File S2). Data may be made available to researchers upon request.

### Haplotype Inference

We estimated the haplotype pair for each animal in the sample using BEAGLE3.3.2 algorithm [20], which models genotype vectors of each individual using a Hidden Markov Model (HMM). The model can be written as follows:
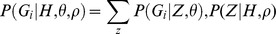



Where:
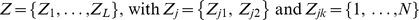



*Z* represents the haplotype pair, for the SNP *j*, originating from a reference panel, which is being copied to form the genotype vector.

*P(* *Z* *|H,ρ)*, is defined by a Markov chain, and it models how the haplotype pair copied from the reference panel changes during the sequence due to a recombination map (*p*) defined throughout all the genome.

*P(G|* *Z*,*θ)*, generates the observed variation of the genotype vectors regarding to the copied haplotypes of the reference panel through a mutation rate (*θ* ) [21].

### Linkage Disequilibrium Computation

The LD coefficient selected for this study was the squared correlation coefficient between pairs of SNPs (*r^2^*) represented as:
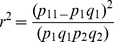
where *p* **_1_** and *p* **_2_** are the minor and major allele frequencies in the SNP1 respectively, *q* **_1_** and *q* **_2_** are the minor and major allele frequencies in the SNP2 respectively, and *p* **_11_** corresponds to the frequency observed between both minor alleles in the same individual throughout the whole population. In addition, a sample size correction was applied to all computed *r^2^* values using the following equation:
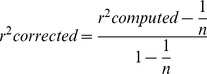
where *n* represents the number of haplotypes in the sample [22]. (For more details see Table S4 in File S4).

### Definition of structural variations based on short range LD

We define a LD-based Structural Variation as follows: first, for each breed and each chromosome we obtain a set of LD means, called expected means, calculating the linkage disequilibrium (*r* **^2^**) of short range (≤100 Kb), sorting SNP pairs by distance, and obtaining the means using bins of 5 Kb. Then we interrogate SNP by SNP looking for segments of at least 1 Kb, consisting of a set of at least 3 adjacent SNPs so that, for each SNP, *r* **^2^** within its neighbors in a 100 Kb range, to the right side of that SNP, are all bigger than, or all smaller than, the corresponding expected means, and their *P*-values from a *t*-test for equality of means are significant after a Benjamini-Hochberg multiple testing correction. In addition, to account just for homogeneously distributed regions consider only SNPs having at least 15 SNP neighbors within 100 Kb.

### Correction for multiple testing

A Benjamini and Hochberg correction for multiple testing [23] was applied to *P*-values in order control the False Discovery Rate. The approach is as follows: first, all *P*-values are sorted from smallest to largest. Denote the *i*-th smallest *P*-value by *p_(i)_*, for each *i* between 1 and *m* (*m* is the total number of *P*-values), then, starting from the largest *P*-value *P*_(*m*)_, compare *P*_(*m*)_ with 0.05 x *i*/*m*. Continue as long as *P*_(*i*)_>0.05 x *i*/*m*. Let *k* be the first time when *P*_(*k*)_ is less than or equal to 0.05 x *k*/*m*, and declare the differences corresponding to the smallest *k P*-values as significant.

### Principal Components Analysis

Vectors constructed with the number of structural variations per chromosome were used to perform a principal component analysis (PCA) and look for differentiation between cattle subgroups. We used R software to perform this analysis. The central idea of PCA is to reduce the dimensionality of a data set which consists of a large number of interrelated variables, while retaining as much as possible of the variation present in the data set. This is achieved by transforming a new set of variables, the principal components (PCs), which are uncorrelated, and which are ordered so that the first few retain most of the variation present in all the original variables [24].

Formally, PCA is defined as an orthogonal linear transformation that transforms the data to a new coordinate system such that the greatest variance by any projection of the data comes to lie on the first coordinate (called the first principal component), the second greatest variance on the second coordinate, and so on. PCA is theoretically the optimum transform for a given data in least square terms. The procedure for obtaining PCAs can be summarized as follows:

Given a vector **X^T^** of *n* dimensions, *X^T^ = [x_1_, x_2_, …, x_n_]^T^*, whose mean vector **M** and covariance **C** are described by:
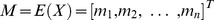


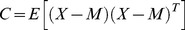



Calculate the eigenvalues λ_1_, λ_2_, …, λ_3_, and the eigenvectors *P_1_*, *P_2_*, …, *P_d_*; arrange them according to their magnitude.
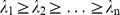



Select *d* eigenvectors to represent the *n* variables, *d*<*n*. Then the *P_1_*, *P_2_*, …, *P_d_* are called the principal components.

## Results

### Polymorphic proportions and Linkage Disequilibrium

**Figure 1:**
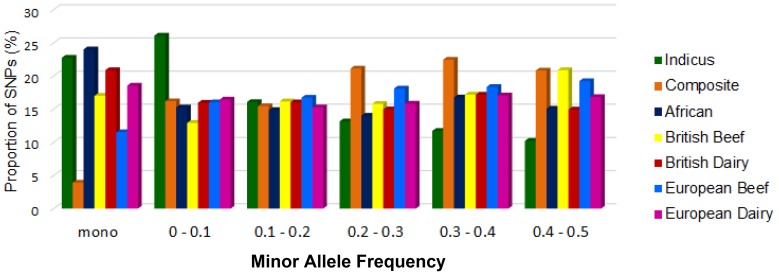
MAF Distribution. Average proportion of SNPs of various frequencies by Cattle group.

The filtered data set included an average of 523,651 genotypes per breed, among the 29 autosomal chromosomes in 506 animals from 19 breeds (See Methods section for a description of quality control filters, Table S2 in File S2 for details of total markers in each breed and chromosomal separately, and Table S1 in File S2 for details on breeds descriptions and sample numbers). To characterize SNPs, we determined the distribution of minor allele frequencies (MAF) in each of the 19 breeds (see Figure 1 and Figures S1 to S7 in Figure S1 for distributions of MAF and Table S3 in File S3 for statistics of MAF).

The lone tropically adapted *Bos taurus taurus* breed, N'Dama and two of the *Bos taurus indicus* breeds, Gir and Nelore, had the lowest polymorphic proportion of SNPs (67%, 71%, and 74%, respectively). The Brahman may have a higher rate of polymorphism than the remaining indicine breeds because that breed was established by importing primarily bulls and backcrossing to taurine derived females. The *Bos taurus taurus* breeds were generally moderately variable with an average of 83% of polymorphic SNPs. The *Bos taurus indicus* breeds show the lowest average polymorphic SNPs with 81%. Figure 1 considers all SNPs (including monomorphic and polymorphic SNPs) but for all subsequent analyses monomorphic SNPs were eliminated from this study because they are uninformative.

**Figure 2:**
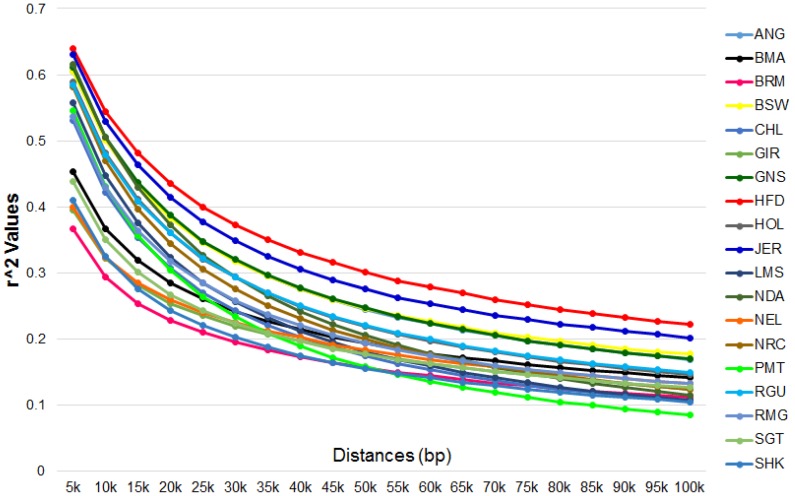
Linkage Disequilibrium. - Genome-wide LD decay in all breeds.

We estimated the haplotype pair for each animal in the sample using Beagle 3.3.2 [20], and calculated the LD for each breed and reported the correlation coefficient, *r* **^2^**. Values of LD were corrected for sample size using the equation described by *Villa-Angulo et al.* (2009) [22] and for each SNP we calculated all the values of LD to a maximum pairwise distance of 100 Kb. The average values were determined using bins of 5 Kb. As observed in figure 2, in short distances (5 Kb) the breed with the lowest average of LD were the *Bos taurus indicus* breeds: Brahman, Gir, and Nelore, with 0.367, 0.395, and 0.401, respectively, while the breeds with the highest average of LD were Hereford, Jersey, and N'Dama, with 0.639, 0.630, and, 0.616, respectively. On the other hand, in large distances (100 Kb), the breeds with the lowest averages of LD were Piedmontese, Sheko, and Charolais, with 0.085, 0.104, and 0.105, respectively, while the breeds with the highest average of LD were Hereford, Jersey, and Brown Swiss, with 0.222, 0.201, and 0.177, respectively. (see Table S4A in File S4 for details)

When comparing these results to values from another study of LD decay with lower density data (BovineSNP50), using the same samples, the trends obtained are quite similar, even when only high density SNPs regions in chromosomes 6, 14, and 25 were considered [22]. It should be noted that [all or most] of the animals used in this study were the same as those in the previous investigation, although the number of marker pairs closer than 20 Kb using the BovineSNP50 was quite small.

### Structural variations

**Figure 3:**
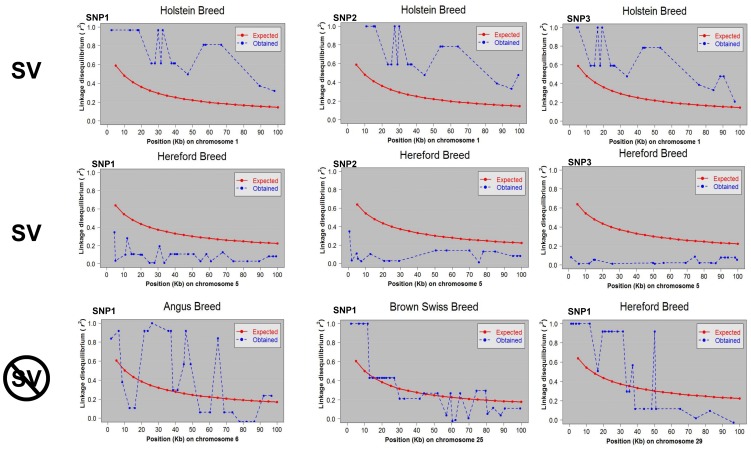
Structural Variation. Two samples declared as structural variations and one declared as containing no structural variation. Red line plots the expected LD. Blue line plots the actual LD between the first SNP and all its neighbors. At the top, three adjacent SNPs are inspected for LD with their neighbor SNPs in a range of 100kb, to the right. For the three SNPs, LD values are bigger than their expected LD mean and were declared significant after multiple testing corrections. In the middle, an example where the LD is smaller than expected LD mean, and as the previous example they were declared significant after correction. At the bottom, an example declared as no structural variation because LD for some of the SNP pairs alternate from being bigger than to smaller than their expected LD mean.

For each breed and each chromosome we obtained a set of LD means (expected means) using bins of 5 Kb. Then, for each SNP we calculated LD with each polymorphic SNP within 100 Kb to the right of that SNP. Putative structural variations were defined where all LD measures were bigger than, or all smaller than, their corresponding expected means, for all SNP pairs within 100 Kb region for at least 3 adjacent polymorphic SNPs. Figure 3 shows two samples declared as structural variants and one declared as containing no structural variants. At the top of the figure; for three SNPs, it is shown LD values with all SNPs within 100 Kb to their right. For these three SNPs, LD values are larger than their expected mean, and were declared significant after multiple testing corrections. In the middle of Figure 3, LD values are shown for another example where *r* ^2^ values are smaller than the expected means, and as the previous example they were declared significant after correction. At the bottom, an example presumed to contain no structural variation because LD for some of the SNP pairs alternate from being bigger than to smaller than their expected LD mean (red line monotonically decreasing).

Using the above definition we scanned all autosomal chromosomes for each breed. Table 1 shows a summary of the structural variations found in the 19 breeds. The total number of variations found among the 19 breeds was 15,246. By grouping all variations across all animals in the sample we defined 9,146 regions. The total number of SNPs involved in the regions was 53,137, representing the 6.40% (160.98 Mb) from the bovine genome (see Table S7 in File S7 for details of chromosome, start position, end position, size in base pairs, number of SNPs, number of breeds, and breeds name involved in each region). Hereford, Red Angus, and Jersey breeds showed the largest number of structural variations, with 1444, 1443, and 1163 respectively, while Nelore, BeefMaster, and Gir showed the smallest number, with 296, 305, and 315 structural variations, respectively (see Table 1). The average distance covered by structural variations genome-wide across all breeds was 15.16 Mb. The largest region declared as structural variation was in the Santa Gertrudis breed, with 396 Kb size. The smallest region was found in Charolais, Gir, Guernsey, Limousine, N'Dama, and Sheko with 1 Kb size. The average of SNPs per structural variation was 6.49 SNPs. The average of variations per Mb genome wide was 0.30, and the average of variations per chromosome was 27.67 (see Table S5 in File S5). The chromosome with the highest average of variations was chromosome 5 with 0.46 variations per Mb, and the chromosome with the smallest average of variations was chromosome 28, with 0.17 variations per Mb.

**Table 1:** Statistics of variations found genome-wide in all breeds.

BREED	No. of SVs	Total Size (Mb)	Average Size (Kb)	Size Max (Kb)	Size Min (Kb)	No. of SNPs	Average SNPs per SV	Max SNPs per SV
ANG	1052	20.55	19.54	295.01	1.11	7676	7.29	93
BMA	305	5.08	16.67	197.73	1.15	1670	5.47	51
BRM	359	6.29	17.52	139.35	1.14	1871	5.2	35
BSW	1093	22.85	20.91	217.88	1.14	8030	7.34	100
CHL	748	12.09	16.16	218.68	1.00	4982	6.66	55
GIR	315	6.08	19.32	209.72	1.00	1756	5.57	41
GNS	1082	22.49	20.79	236.56	1.00	8044	7.43	62
HFD	1444	31.05	21.50	271.71	1.07	11363	7.86	87
HOL	855	14.15	16.56	220.68	1.28	5391	6.30	70
JER	1163	25.31	21.76	319.75	1.15	9001	7.73	102
LMS	849	13.08	15.41	191.31	1.00	5236	6.16	70
NEL	296	5.30	17.93	122.57	1.50	1560	5.27	29
NRC	1051	18.46	17.56	153.62	1.09	7223	6.87	62
NDA	1060	20.80	19.63	194.13	1.00	7242	6.83	48
PMT	727	10.82	14.89	166.70	1.06	4544	6.25	77
RGU	1443	29.59	20.50	280.74	1.06	11075	7.67	138
RMG	728	12.13	16.66	178.72	1.23	4715	6.47	78
SGT	336	6.39	19.02	396.03	1.17	1992	5.92	79
SHK	340	5.61	16.50	190.78	1.00	1733	5.09	38
**TOTAL AVE.**	**802.42**	**15.16**	**18.35**	**221.14**	**1.11**	**5531.78**	**6.49**	**69.21**

From Table 1 we can observe that the breeds with the highest number of SNPs falling in SVs (from now and on we abbreviate structural variation as SV) were Hereford with 11363, Red Angus with 11075, Jersey with 9001, Guernsey with 8044, Brown Swiss with 8030 and Angus with 7676, all from the *Bos taurus* group. While, Nelore, BeefMaster, Sheko, Gir, Brahman and Santa Gertrudis were the breeds with the smallest number of SNPs falling in SVs, with 1560, 1670, 1733, 1756, 1871, and 1992 respectively. All these breeds, are from *Bos indicus* and *composite* groups. The average number of SNPs falling in SVs across all breeds was 5531.78. The breeds with the highest average of SNPs per SV were Hereford, Jersey, Red Angus, Guernsey, Brown Swiss, Angus, and Norwegian Red, all from the *Bos taurus* group, while the breeds with the smallest average of SNPs per SV were Sheko, Brahman, Nelore, BeefMaster, Gir, and Santa Gertrudis, from the *Bos indicus* and composite groups.

The 5 breeds with SVs containing the largest number of SNPs (last column in Table 1) were Red Angus, Jersey Brown Swiss, Angus and Hereford, all from the *Bos taurus* group. Asian breeds resulted with the smallest SV, in SNPs quantity.

**Figure 4:**
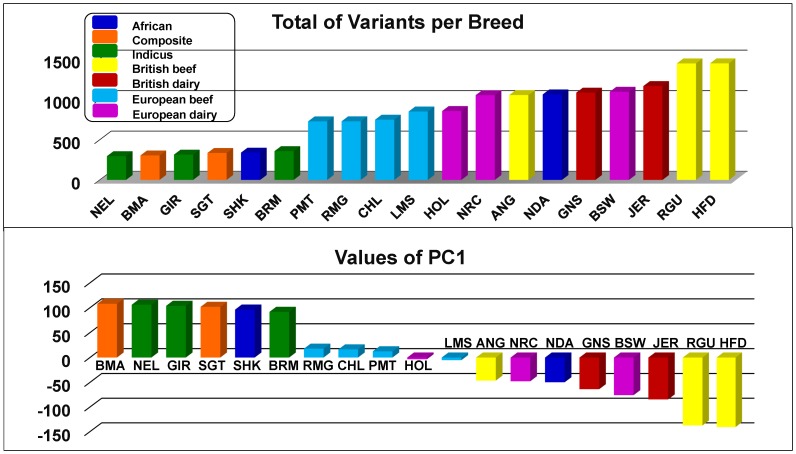
Number of structural variations per breed. Genome wide averaged number of structural variations per breed.

According to the number of proposed structural variants for each breed, shown in Figure 4, Hereford breed has the highest number of variations, while Nelore has the smallest. There is over a three-fold difference among the breeds represented in this study. Even if there are artifacts associated with genome assembly errors there is still evidence for immense breed differences in these results.

## Discussion

**Figure 5:**
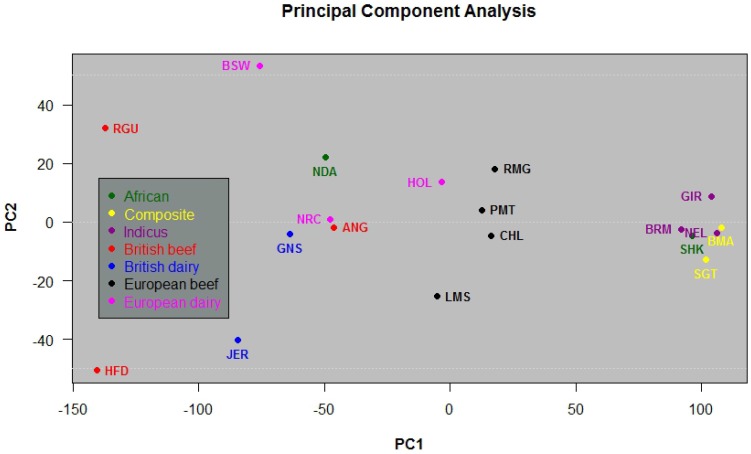
Principal Component Analysis of structural variation statistics. PCA of the number of structural variations found per chromosome per breed.

In order to investigate the closeness in variability among breeds given the amount of identified structural variations; for each breed we constructed a vector of 29 fields, where each field contained the number of SVs in a chromosome. Principal Component Analysis [24] was applied to this vectors. The results are displayed in figure 5, where we observe how some breeds show closeness given the geographic region of evolution. Beef European breeds: Piedmontese, Charolais, Romagnola and Limousin, for example, appear really close, possible reflecting the geographic closeness of evolution regions, Italy and France. Asian breeds (*Bos indicus*): Gir, Brahman and Nelore resulted with positive loads if observed from PC1 and they are displayed really close. In the same way composite breeds: Santa Gertrudis and BeefMaster resulted with positive loads (for PC1), and they are displayed really close too. Some British breeds: Angus, Red Angus, Hereford, Jersey, and Guernsey, and European breeds: Brown Swiss, Norwegian Red and Holstein, all resulted with negative loads, when observed from PC1 axis, and appear relatively separated from the other groups. On the other hand, the two breeds from the African group, N'Dama and Sheko, appear relatively far from each other. It is explained from the fact that Sheko is a *Taurus Indicus* breed while N'Dama is *Taurus Taurus*.

In support of our results that show a clear regional differentiation between breed groups given the quantity of variations, we analyzed the result of a study that was carried out in the “Y” chromosome of European domestic cattle [25], Authors found a noticeable separation of a set of haplotypes called Y1, which appear with a high frequency in cattle breeds located on the Northwestern region of Europe and the British isles, and another set of haplotypes called Y2, with a dominant character on the cattle located at the Southern region of Europe.

### Comparison with other reported structural variations

**Figure 6:**
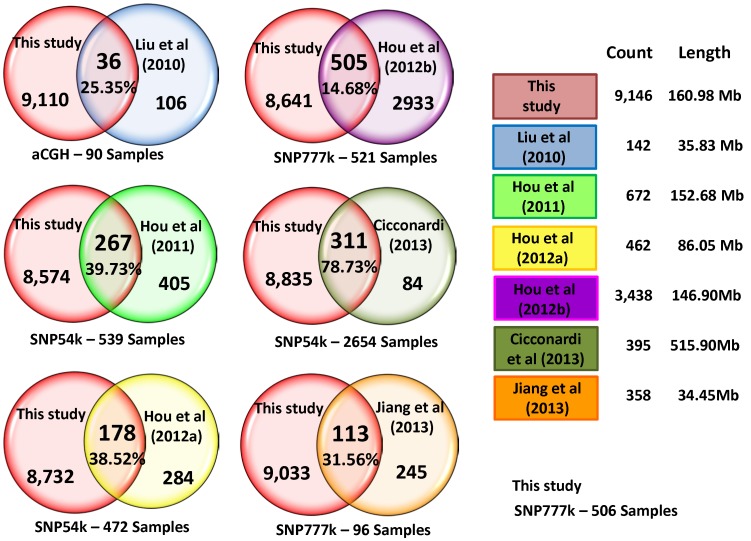
Comparison of LD-based structural variations to other reported variations. LD-based structural variations compared to other studies reported variations.

Given that we found 6.4% of the genome involved with SV, we intuitively suggested overlapping with other types of variations already reported by other groups. Then, we analyzed how much overlap existed between our findings and structural variations from the CNV type. We compared the number, position, and length of structural variations. Figure 6 presents a comparison of our results with six studies on CNVs reported by *Jiang et al.* (2013) [26], *Cicconardi et al.* (2013) [11], *Hou et al*. (2011, 2012a and 2012b) [13], [14], [27] and *Liu et al.* (2010) [16] The first three studies considered just Holstein and Angus breeds. For comparison we considered their CNV regions only in autosomal chromosomes. For *Jiang et al* (2013) we found 113 overlaps of 358 CNVRs, representing the 31.56%. For *Cicconardi et al.* (2013), we found 311 overlapping regions, representing the 78.73% of the 395 reported regions. For *Hou et al* (2012a, 2012b, 2011), we found 178, 505 and 267 overlaps of 462, 3438 and 672 variations reported, representing the 38.52%, 14.68% and 39.73% of overlapping regions respectively. Finally for *Liu et al.* (2010), we considered just the eleven breeds both studies have in common. We found 36 overlapping regions, representing the 25.35% of the 142 reported regions.

Next, we searched in NCBI [28], for genes involved in our defined structural variations. The largest number of genes was found in Red Angus, Hereford and Jersey breeds, with 490, 487 and 379 genes, respectively. The smallest number of genes was found in BeefMaster, Nelore and Santa Gertrudis breeds, with 105, 115 and 126 genes, respectively. (For complete details see Table S6A in File S6 for chromosome number, start position, end position, size in base pairs, name of gene, type of gene, and gene description; Table S6B in File S6 for the number of genes per breed; and, Table S6C in File S6 for the number of genes involved with structural variations in each chromosome).

The last analysis was to look within our defined structural variations for regions that were consistent in all 19 breeds. We did not found common regions among all breeds. However, there are 2 regions where 17 breeds coincided, 2 regions where 15 breeds coincided, 2 regions where 14 breeds coincided, 3 regions where 13 breeds coincided and 13 regions where 12 breeds coincided, even when they do not coincide with the total length, at least they share one segment declared as variation. (For complete details see Table S7 in File S7).

## Conclusions

In this work we present a simple definition of genomic structural variation based on the deviation of the expected short range LD between SNPs. Using this definition we performed a genome-wide analysis in Cattle. The total number of variations found among 19 breeds was 15,246. By grouping all variations across all animals in the sample we defined 9,146 regions. The total number of SNPs involved in the regions was 53,137, representing the 6.40% (160.98 Mb) from the bovine genome. The number of genes covered by these variations was 3,109. From a comparison of overlapping with variations of the CNV type previously reported we found overlapping ranging from 14.68% to 78.73%,

An analysis of differentiation based on the number of structural variations genome-wide showed great difference between some breeds and reveled closeness of groups given the geographic region in which they are evolving. Finally, even when there is overlapping of LD-based Structural Variations with CNVs, they capture different genomic variation patterns, and further studies would be necessary in order to elucidate their association to disease and other phenotypic traits.

## Supporting Information

Figure S1 **Figures S1–S7: MAF Distribution.** Average proportions of SNPs of various frequencies per breed and per groups.
(DOCX)

File S1 **Table S1.** Breeds and number of animals in the sample.
(XLSX)

File S2 **Table S2.** Final summary of markers for our study.
(XLSX)

File S3 **Table S3.** Mean and Median MAF average of 19 breeds.
(XLSX)

File S4 **Table S4.** Average values obtained by *r^2^* for all breeds (LD decay).
(XLSX)

File S5 **Table S5.** Variants per Chromosome and average per Megabase.
(XLSX)

File S6 **Table S6A.** Type, name and description of genes found in this study. **Table S6B**: Number of genes per breeds. **Table S6C**: Number of genes per chromosome.
(XLSX)

File S7 **Table S7.** Variation Structural Regions.
(XLSX)
