# Supplementary material for: High Density LD-Based Structural Variations Analysis in Cattle Genome
Source: PLoS One. 2014 Jul 22;9(7):e103046. doi: 10.1371/journal.pone.0103046 (PMC4106904; doi:10.1371/journal.pone.0103046)
Supplement: Figure S1 — Figures S1–S7: MAF Distribution. Average proportions of SNPs of various frequencies per breed and per groups. (DOCX) [file pone.0103046.s001.docx]

**Figure S1.- MAF Distribution.** Average proportions of SNPs of various frequencies per breed.


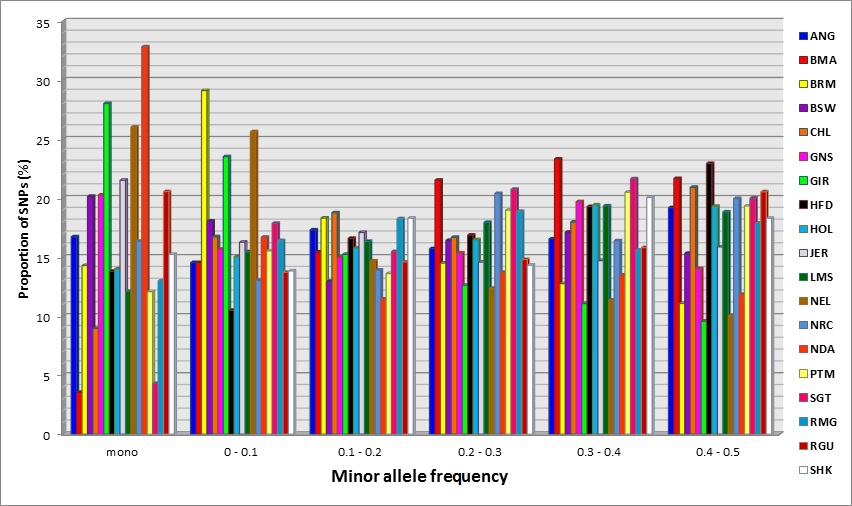


|  |
| --- |

**Figure S2.- MAF Distribution.** Average proportions of SNPs of various frequencies of group African.


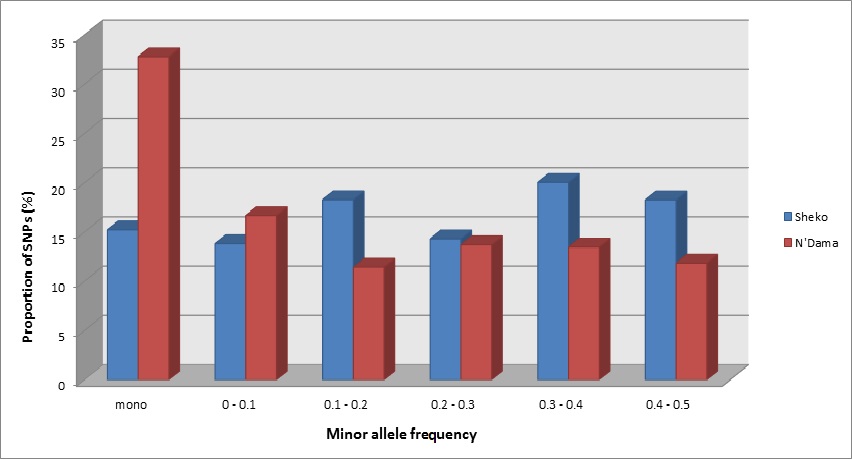


|  |
| --- |

**Figure S3.***-* **MAF Distribution.** Average proportions of SNPs of various frequencies of group Indicus.


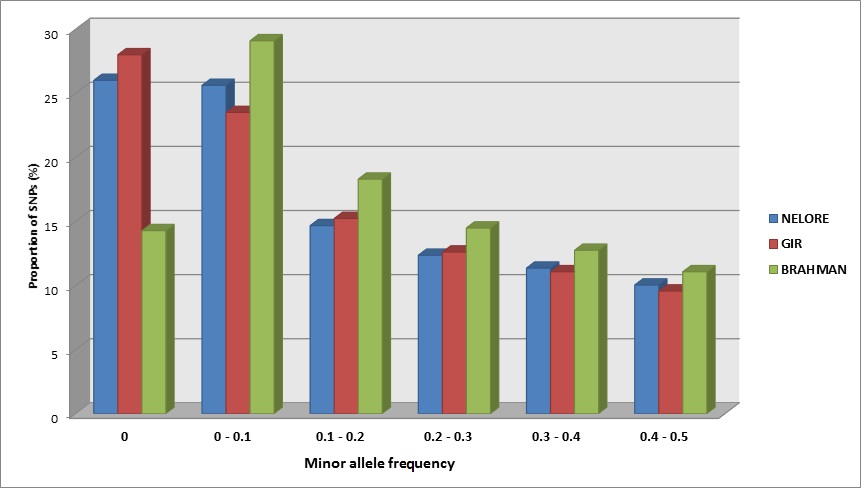


|  |
| --- |

**Figure S4.***-* **MAF Distribution.** Average proportions of SNPs of various frequencies of group Composite.

|  |
| --- |

**Figure S5.***-* **MAF Distribution.** Average proportions of SNPs of various frequencies of group Beef.

|  |
| --- |

**Figure S6.***-* **MAF Distribution.** Average proportions of SNPs of various frequencies of group Dairy.

|  |
| --- |

**Figure S7.***-* **MAF Distribution.** Average proportions of SNPs of various frequencies by breed.

| **MAF** | **ANG** | **BMA** | **BRM** | **BSW** | **CHL** | **GNS** | **GIR** | **HFD** | **HOL** | **JER** | **LMS** | **NEL** | **NRC** | **NDA** | **PTM** | **SGT** | **RMG** | **RGU** | **SHK** |
| --- | --- | --- | --- | --- | --- | --- | --- | --- | --- | --- | --- | --- | --- | --- | --- | --- | --- | --- | --- |
| **0 - 0.1** | 14.53 | 14.53 | 29.09 | 18.05 | 16.72 | 15.66 | 23.5 | 10.5 | 15.03 | 16.26 | 15.46 | 25.61 | 13.03 | 16.68 | 15.52 | 17.86 | 16.4 | 13.72 | 13.82 |
| **0.1 - 0.2** | 17.3 | 15.43 | 18.3 | 12.95 | 18.75 | 14.99 | 15.23 | 16.59 | 15.77 | 17.08 | 16.33 | 14.67 | 13.9 | 11.44 | 13.6 | 15.47 | 18.25 | 14.58 | 18.29 |
| **0.2 - 0.3** | 15.71 | 21.51 | 14.49 | 16.41 | 16.67 | 15.35 | 12.61 | 16.86 | 16.47 | 14.57 | 17.94 | 12.36 | 20.38 | 13.71 | 18.96 | 20.74 | 18.87 | 14.8 | 14.31 |
| **0.3 - 0.4** | 16.53 | 23.3 | 12.75 | 17.11 | 17.97 | 19.69 | 11.07 | 19.28 | 19.41 | 14.7 | 19.33 | 11.36 | 16.38 | 13.48 | 20.49 | 21.62 | 15.59 | 15.8 | 20.04 |
| **0.4 - 0.5** | 19.18 | 21.65 | 11.07 | 15.32 | 20.9 | 14.02 | 9.57 | 22.93 | 19.3 | 15.86 | 18.82 | 10.04 | 19.95 | 11.85 | 19.32 | 19.99 | 17.88 | 20.53 | 18.28 |
|  | **83** | **96** | **86** | **80** | **91** | **80** | **71** | **86** | **86** | **78** | **88** | **74** | **84** | **67** | **88** | **96** | **87** | **79** | **85** |

|  |
| --- |
